# Canine medial retropharyngeal lymph node measurements on T2 spin-echo sequences at 3T

**DOI:** 10.3389/fvets.2024.1506670

**Published:** 2024-12-09

**Authors:** Emily B. DuPont, Elizabeth Boudreau

**Affiliations:** School of Veterinary Medicine and Biomedical Sciences, Department of Small Animal Clinical Sciences, Texas A&M University, College Station, TX, United States

**Keywords:** cervical, MUO, glymphatic, magnetic resonance imaging, dog

## Abstract

**Introduction:**

The objective of this study is to estimate reference values for medial retropharyngeal lymph nodes (MRLNs) measured in high-field (3T) MRI studies of the canine head/brain using transverse T2 spin-echo images and to determine if dogs with structural brain disease exhibit medial retropharyngeal lymph nodes that are larger than expected from estimated reference values.

**Methods:**

The study population comprises 142 MRLNs from 71 dogs with no evidence of structural brain disease and normal CSF evaluation and 116 MRLNs from 58 dogs with structural brain disease confirmed by histopathology as of infectious or neoplastic origin, or to represent meningoencephalitis of unknown etiology.

**Results:**

Based on this sample, MRLNs are expected to measure 2.9–12.4 mm in maximum short-axis transverse diameter. Interobserver measurement differences are ~1 mm in 95% of the sampled subjects. Lymph node size is correlated with body weight (*R* = 0.47–0.52) and age (*R* = −0.39 – −0.47).

**Discussion:**

No difference was found between the lymph node size of dogs with structural brain disease of any type, or overall, compared to that of dogs without structural brain disease.

## 1 Introduction

**Figure 1:**
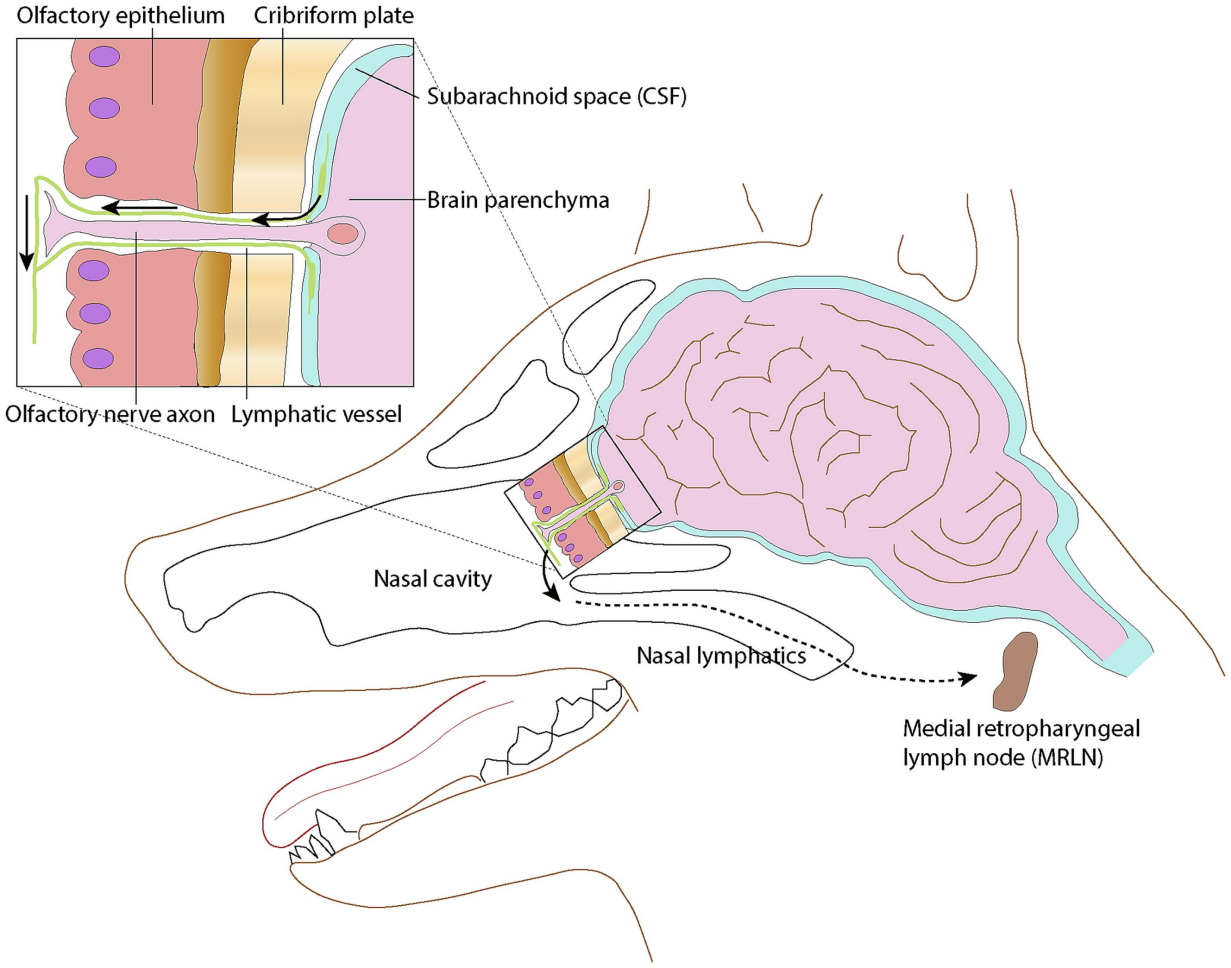
Schematic representation of the lymphatic drainage pathway via the cribriform plate into the nasal lymphatics. Lymphatic vessels surround olfactory nerve cell axons as they penetrate the cribriform plate, then merge with lymphatic vessels in the submucosa of the caudal nasal cavity. Modified from (1); licensed under CC BY 4.0.

The idea of lymphatic vessels contributing to the drainage of cerebrospinal fluid (CSF) from the central nervous system has been a topic of ongoing investigation over the past few decades. It has been demonstrated that across numerous mammalian species, CSF exits the central nervous system to enter the lymphatics present in the nasal submucosa (Figure 1) (1, 2). This pattern remained constant among the species studied, suggesting it could be a feature in all mammals. In the dog, the tissues of the head, including the nasal cavity, drain in part via lymphatic vessels to the retropharyngeal lymph center (3). It has been shown experimentally that fluid injection into the subarachnoid space of a dog results in increased cervical lymphatic drainage (4).

The retropharyngeal lymph center in the dog is most consistently represented by the medial retropharyngeal lymph node (MRLN), which also serves as the collective terminal for drainage from midline structures of the head, including the nasal cavity.

Given the evidence of drainage of the CSF from the central nervous system to the nasal lymphatics and the known pathway from the nasal lymphatics to the retropharyngeal lymph center, it can be suspected that there may be a connection between the CSF and the retropharyngeal lymph center. There has been speculation that disease of the central nervous system may impact the size or appearance of the MRLNs due to this supposed connection. Although previous studies have investigated the size of MRLNs in dogs using the modalities of ultrasound, CT, and MRI, as well as their characteristics in various head and neck diseases, their appearance and size in the context of central nervous system diseases have not been extensively studied.

The medial retropharyngeal lymph node can be imaged during MRI studies of the brain and cranial cervical spine; however, they are not routinely or intentionally included, and there have been few studies investigating the appearance or size of the medial retropharyngeal lymph node using MRI in dogs. To the authors’ knowledge, there have been no studies directly comparing the MRI appearance of medial retropharyngeal lymph nodes of neurologically normal dogs to dogs with structural brain disease. In a study looking at the appearance of inflammatory vs. neoplastic medial retropharyngeal lymph nodes in dogs and cats, neoplastic lymph nodes were found to often be bigger in size than those with inflammatory disease (5). The possibility of using the size of the medial retropharyngeal lymph nodes as an aid in suspecting potential etiologies for structural brain disease remains a topic in need of further investigation.

This study aimed to first summarize the size of the medial retropharyngeal lymph nodes in normal dogs without evidence of structural brain disease and second to determine if their size was increased in dogs with structural brain disease. The hypothesis was that there could be enlargement of the medial retropharyngeal lymph nodes with neoplastic, infectious, or immune-mediated diseases of the central nervous system, likely due to reactivity from drainage of the CSF.

## 2 Materials and methods

### 2.1 Case selection

This was a single-institution retrospective study evaluating canine brain MRIs performed on a single 3T MRI^1^ over 12 years (2011–2023). Identified subjects were required to have transverse T2-weighted spin-echo images without fat suppression (TR: 3500–8,000 ms; TE: 85–96 ms; slice thickness 2.5–3.0 mm) that included both left and right MRLNs at their maximal transverse diameter.

Control subjects were defined as dogs with finalized MRI interpretations that did not identify any structural abnormalities of the imaged region and a normal cerebrospinal fluid analysis, defined as ≤ 5 nucleated cells/*μ*L, ≤ 30*μ*g/dL protein, and hemodilution ≤ 500 RBC/*μ*L.

Affected subjects were required to have necropsy or biopsy-confirmed diagnoses and were categorized into one of the three etiological groups: infectious (INF), meningoencephalitis of unknown etiology (MUO), or primary CNS neoplasia (NEO).

### 2.2 Lymph node measurement

**Figure 2:**
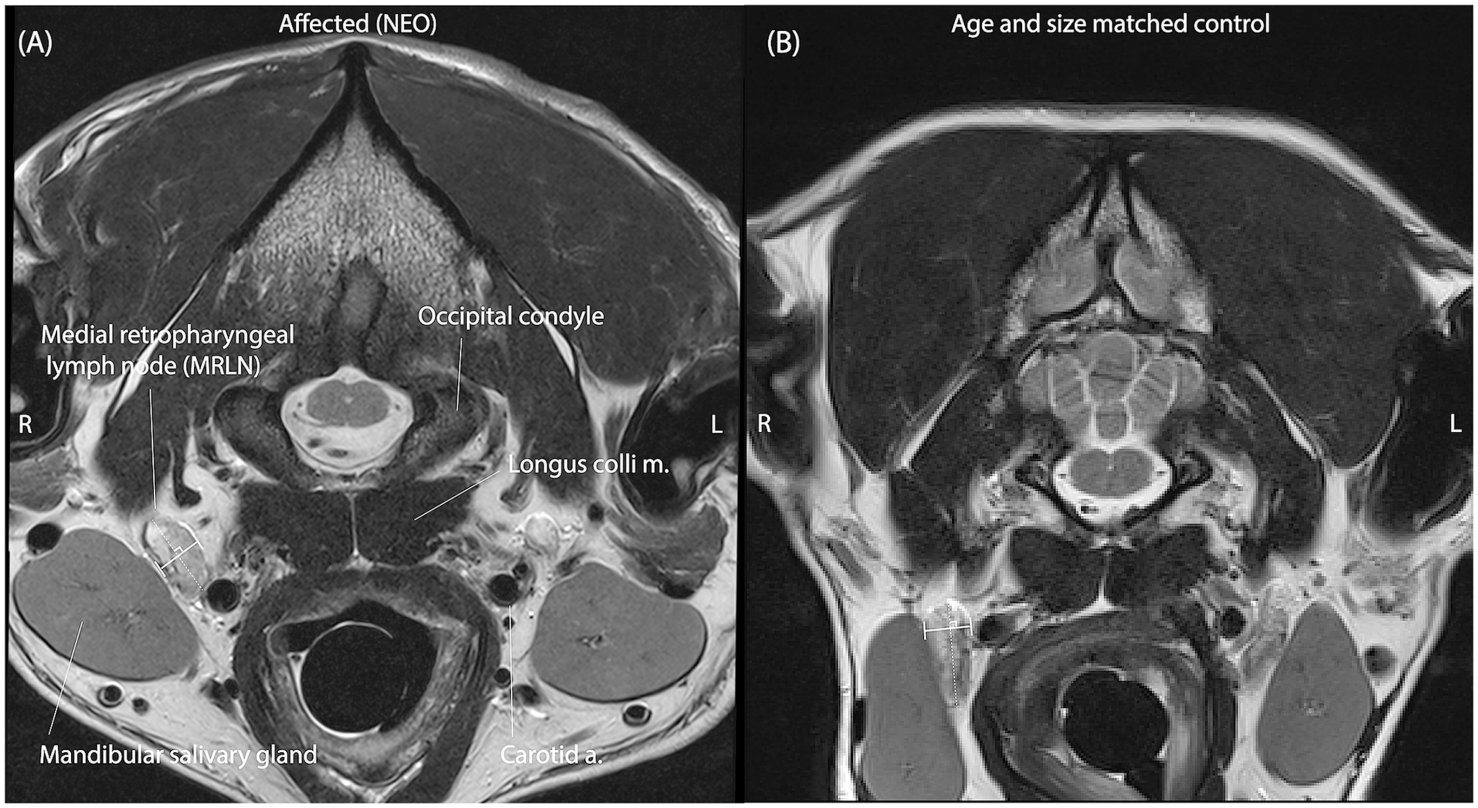
Representative T2 spin-echo slices are selected by both observers independently to represent the M_SATD of the right medial retropharyngeal lymph node in two different dogs. **(A)** A dog with intracranial neoplasia (affected subgroup NEO). **(B)** A dog of similar size and age from the control group. Anatomical landmarks for identification of the MRLN are labeled in panel A. In each panel, the solid white line between perpendicular shorter white lines illustrates the selected short-axis transverse diameter of the lymph node for the represented slice.

MRLNs were identified by their location medial to the mandibular salivary gland and lateral to the common carotid artery near the level of the atlanto-occipital joint (Figure 2) (6). Two observers blinded to all clinical information viewed only the T2 transverse spin-echo series and identified slices representing the maximum short-axis transverse diameter (M_SATD) of the left and right MRLNs independently. In cases where more than one MRLN was identified on a side, only the larger one was recorded. For both the left and right sides, each observer used a caliper to estimate the M_SATD in millimeters (7).

### 2.3 Statistical analysis

Descriptive statistics, histograms, correlation coefficients, and Bland–Altman plots were generated using MATLAB.^2^ A Shapiro–Wilk test was used to evaluate normality. A 95% confidence interval for the difference in observer measurements is reported as quantiles. Intra-class correlation coefficients and 95% confidence intervals were calculated using MedCalc.^3^ For the estimation of reference intervals, the Clinical & Laboratory Standards Institute (CLSI) guidelines (8) were followed as instantiated in MedCalc (see text footnote 3). The averages of the two observers’ measurements were Box–Cox transformed with lambda estimated from each data set. A Shapiro–Wilk test was used again to evaluate normality, and Tukey’s test was used to identify outliers. The robust method with 10,000 Boostrap iterations was used to calculate the 90% CI for the upper and lower limits of the reference intervals. General mixed-effects linear regression was performed on log-transformed control measurements in MATLAB (see text footnote 2) using the Statistics and Machine Learning Toolbox function fitglme, with age and weight as fixed effects variables and side (left or right), observer, and subject as random effects variables. The residuals were visually inspected for outliers, and the model was refit after their removal.

A Kruskal–Wallis test, as instantiated in MATLAB (see text footnote 2), was used to evaluate for differences in age and weight between affected subgroups. Repeated-measures ANOVA, as instantiated in MATLAB (see text footnote 2), on rank-transformed data, was used to evaluate the effect of the etiological group (INF, MUO, or NEO) on the difference between measured lymph node size AND the size predicted by the linear regression model established from the control data. A *p*-value of <0.05 was used to reject null hypotheses (Figures 1, 2).

## 3 Results

### 3.1 Control subjects

**Figure 3:**
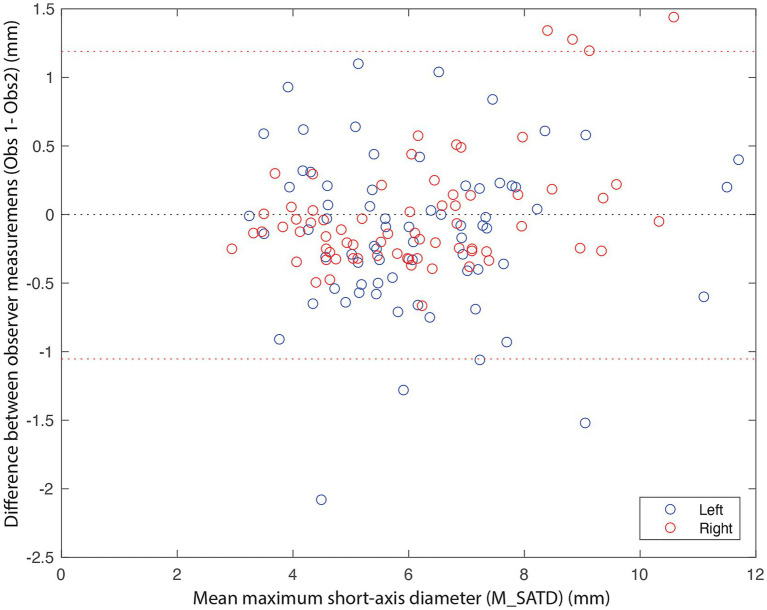
Bland–Altman plot of M_SATD measurements by two observers for the left (blue circles) and right (red circles) medial retropharyngeal lymph nodes of all control cases (*n* = 142). The red dotted lines indicate the 2.5 and 97.5% quantiles of differences between the measurements of the two observers. The black dotted line indicates 0.

**Figure 4:**
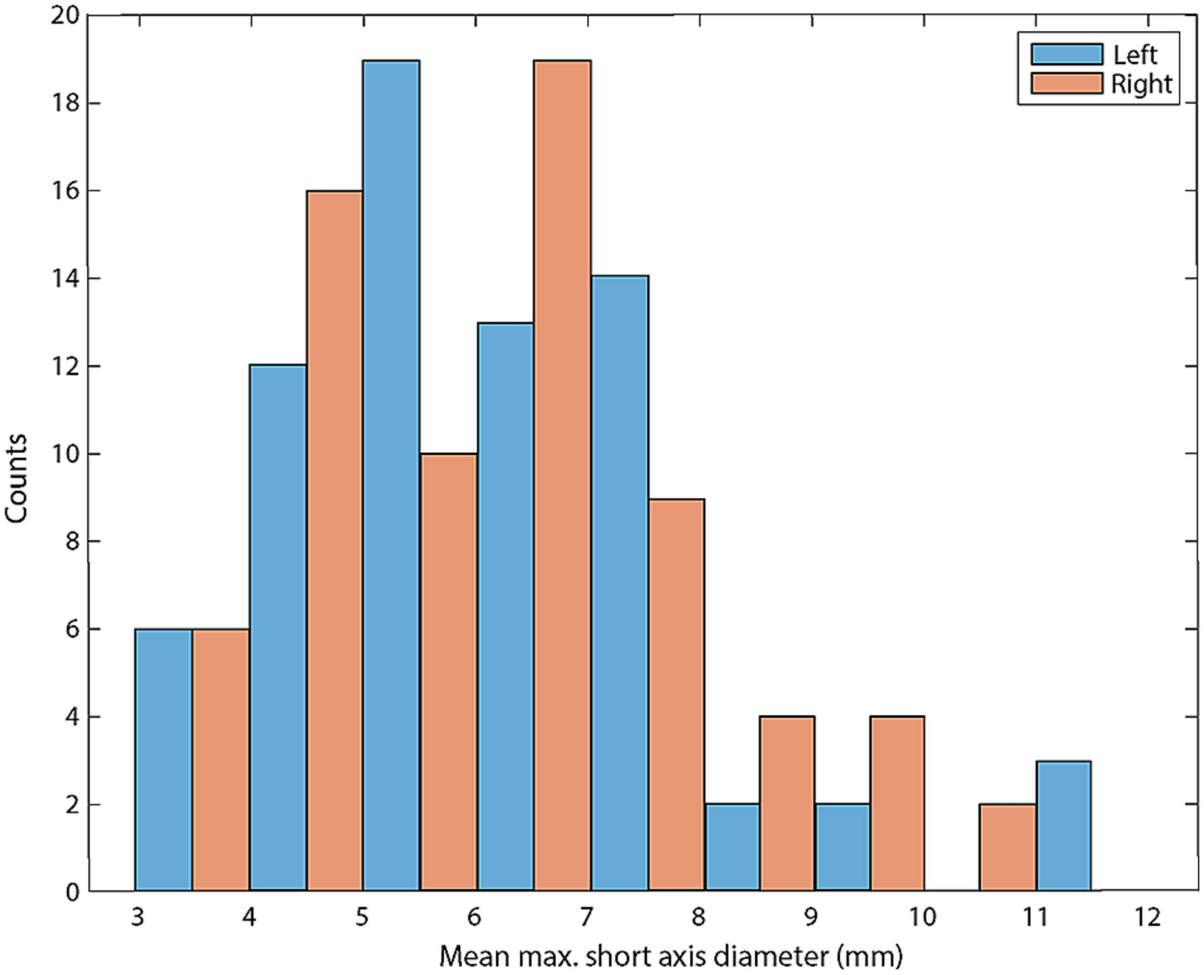
Histogram of mean M_SATD for each control lymph node (in mm; *n* = 142). Blue bars indicate left medial retropharyngeal lymph nodes and red bars indicate right medial retropharyngeal lymph nodes.

In total, 71 control subjects were identified. The median age of the control subjects was 6 years (1–13), and the median weight was 22 kg (2.1–72.6). A Bland–Altman plot was used to qualitatively assess inter-observer agreement for the measurement of M_SATD (Figure 3). The 95% CI for the difference in measurements between the two observers was [−1.0–1.2 mm]. The intraclass coefficient for the two observers was 0.92 (95% CI: 0.89–0.94). The distribution of the average of the two observers’ measurements for the control sample is shown in Figure 4.

**Figure 5:**
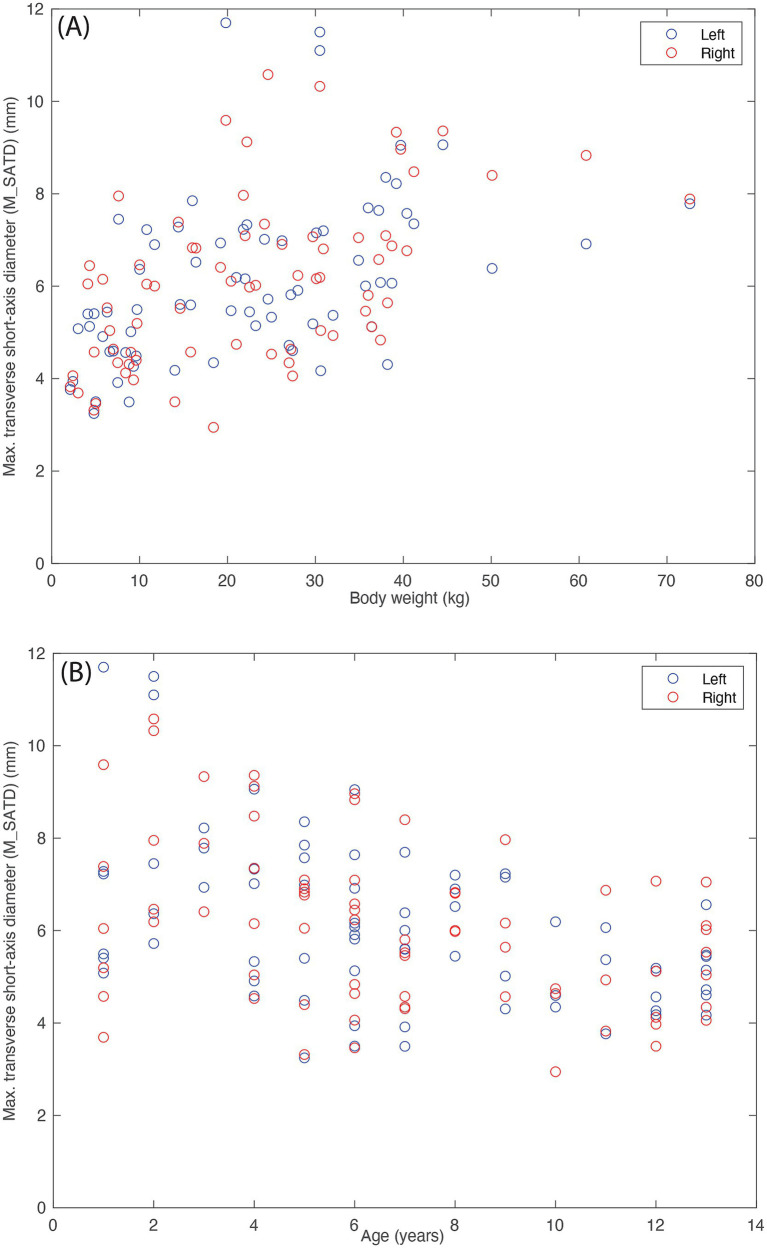
Lymph node size (average of the measurements of the two observers). **(A)** As a function of body weight (in kg), and **(B)** as a function of age (in years) for control subjects (*n* = 142). Blue circles indicate left medial retropharyngeal lymph nodes, and red circles indicate right medial retropharyngeal lymph nodes.

The average of the two observers’ measurements was modestly correlated with weight (Figure 5A; R_left_ = 0.47 and R_right_ = 0.52) and anti-correlated with age (Figure 5B; R_left_ = −0.47 and R_right_ = −0.39). Based on these average measurements, reference intervals were created for left and right MRLN as described above (Table 1). Transformed data were normally distributed, and no outliers were identified.

**Table 1:** Reference ranges with 90% CI of the lower and upper bound for left and right MRLN in control subjects (*n* = 71 for left and *n* = 71 for right).

Reference ranges for left and right medial retropharyngeal lymph node M_SATD in control subjects (mm)				
	Lower bound	90% CI	Upper bound	90% CI
Left	3.5	3.2–3.8	11	9.9–12.4
Right	3.2	2.9–3.6	10.1	9.3–10.9

**Figure 6:**
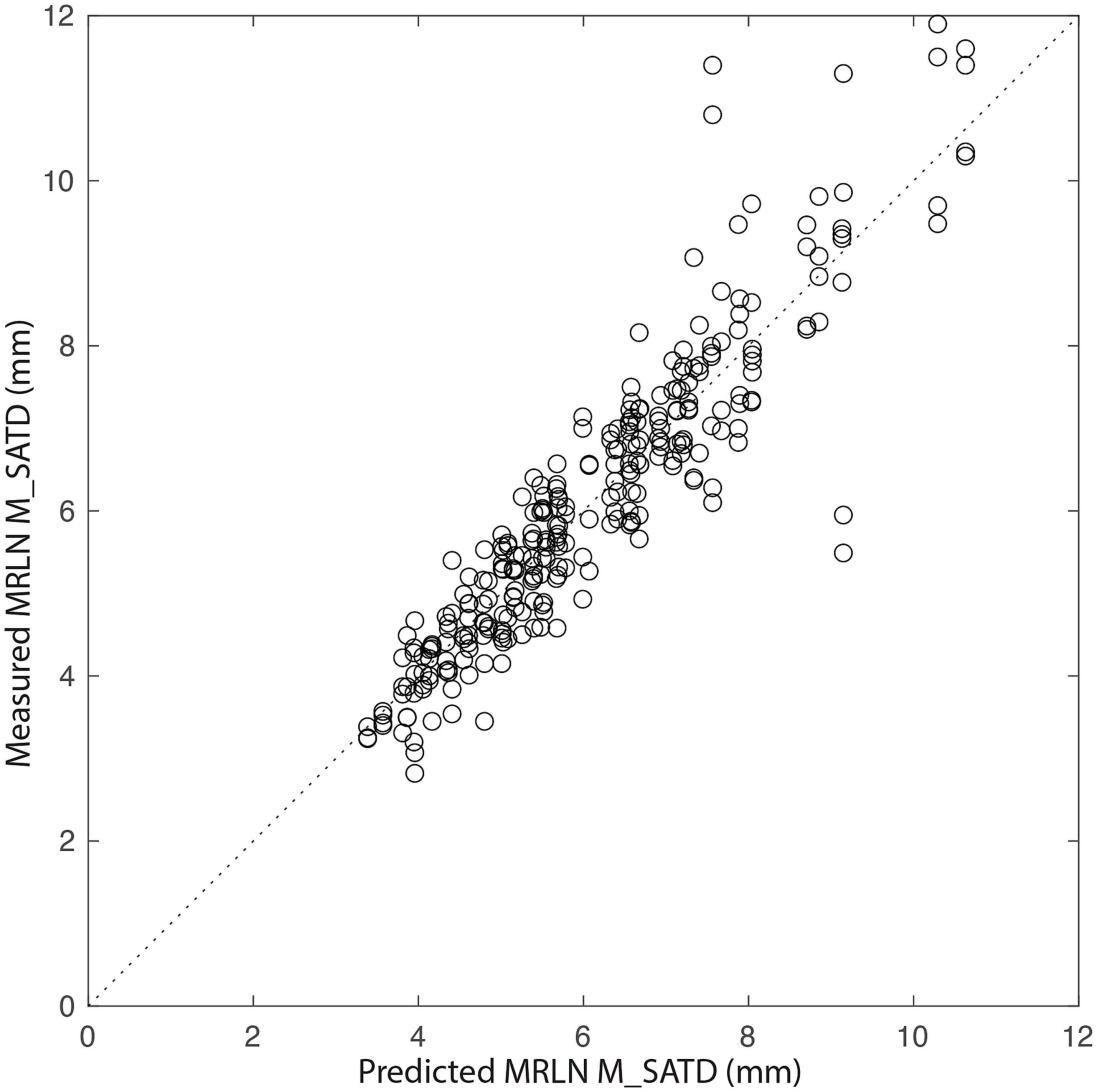
Measured MRLN size vs. that predicted by the generalized linear mixed-effects model. The dotted line represents y = x. Each circle is a single measurement for a single MRLN (left or right) for a single observer (*n* = 284).

To further evaluate the relationship of body weight and age to lymph node size, we created a general linear mixed-effects regression model for lymph node size based on subject age and weight using control data, as described above. The initial model was refit after the removal of outliers (9/284 values). In the final model, residuals were normally distributed, and *R*^2^ = 0.90. Figure 6 shows the relationship between measured lymph node size and that predicted by the final model. The largest lymph node sizes are not well-predicted by the model, with a bias toward underestimation.

**Figure 7:**
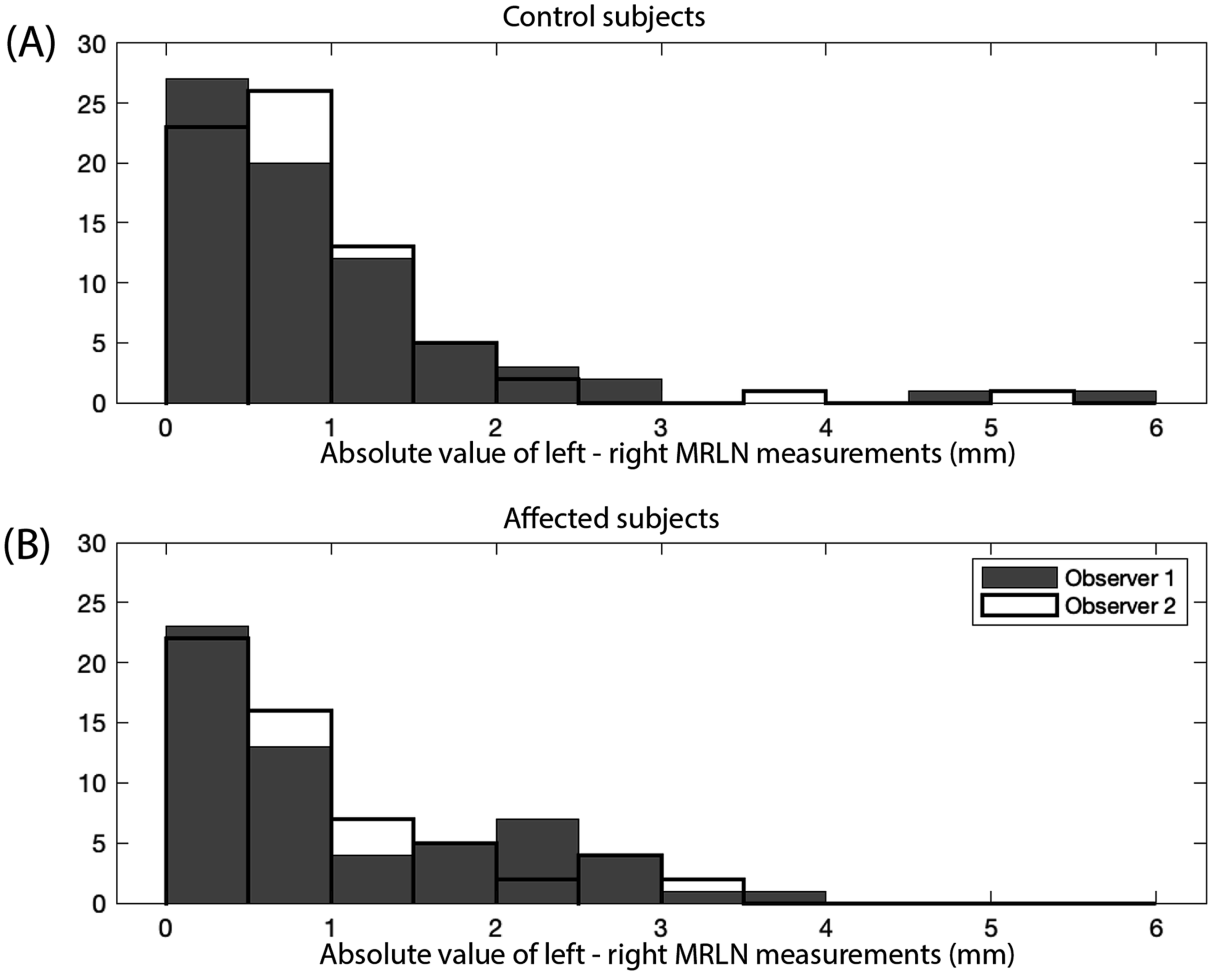
Absolute value of the difference between left and right MRLN measurements (mm). Measurements for observer 1 are shown as gray bars, and measurements for observer 2 are shown as black outlined bars. **(A)** Control subjects (*n* = 142), and **(B)** affected subjects (*n* = 116).

Left–right asymmetry for the MRLNs was generally small (Figure 7A). The median difference between left and right MRLNs was 0.68 mm (range 0.05–4.9) based on average measurements for the control subjects.

### 3.2 Affect subjects

In total, 58 affected subjects were identified. These comprised 10 subjects with infectious disease (INF), 21 subjects with meningoencephalitis of unknown origin (MUO), and 27 subjects with primary nervous system neoplasia (NEO) (Table 2). The median age of the affected subjects was 6 years (1–13), and the median weight was 22 kg (2.1–54.5). For the subgroups, the median age of INF was 3.5 years (1–11), and the median weight was 26.8 kg (9.2–39.5). The median age of MUO was 5 years (1–12), and the median weight was 9.2 kg (2.1–33.6). The median age of NEO was 8 years (3–13), and the median weight was 29.1 kg (4.2–54.5). Dogs with infectious brain disease or MUO, were younger than those with primary CNS neoplasia (*p* = 0.02). Dogs with MUO were smaller than those with infectious brain disease or CNS neoplasia (*p* < 10^−3^).

**Table 2:** Diagnoses for affected subjects in each subcategory (MUO, INF, and NEO), total *n* = 58.

Specific diagnoses for affected subjects		
	Diagnosis	Counts
MUO (*n* = 21)	Necrotizing meningoencephalitis	5
	Granulomatous meningoencephalitis	7
	Undefined meningoencephalitis	9
INF (*n* = 10)	Fungal	6
	Protozoal	2
	Viral	1
	Bacterial (abscess)	1
NEO (*n* = 27)	Meningioma	11
	Glioma	13
	Choroid plexus papilloma	2
	Malignant peripheral nerve sheath tumor	1

Lymph node measurements are again reported as the average of the measurements of two observers. The median and range of lymph node size for each affected subgroup are shown in Table 3. Asymmetry between left and right MRLN was similar between affected subjects and control subjects (Figure 7B, as compared to Figure 7A). The median difference between left and right MRLNs was 0.23 mm (range 0–1.26) based on average measurements for the affected subjects.

**Table 3:** Median and range for lymph node size (average measurements of the two observers) for affected subgroups (MUO, INF, and NEO) and predictions of same based on a linear regression model created from control subject data.

Measured and predicted M_SATD in affected subjects (mm)							
		Median		Min		Max	
		Measured	Predicted	Measured	Predicted	Measured	Predicted
MUO (*n* = 21)	Left	6	5.5	4.6	4.1	9.1	8.9
	Right	6.1	5.7	4.5	4.1	9.1	8.6
INF (*n* = 10)	Left	8	7.5	4.9	5.5	10.8	8.9
	Right	8	7.7	5.2	4.7	10.8	8.6
NEO (*n* = 27)	Left	6.1	6.1	3.2	4.1	10.4	8.9
	Right	6	5.9	3.3	4.1	9.6	8.6

### 3.3 Comparison of affected subjects to expected lymph node size based on control subjects

For each affected subject, the average M_SATD of the left and right MRLNs, as measured by the two observers, was compared to the reference ranges established for the control data set. No lymph nodes were larger than the upper 90% CI of the estimated reference range.

**Figure 8:**
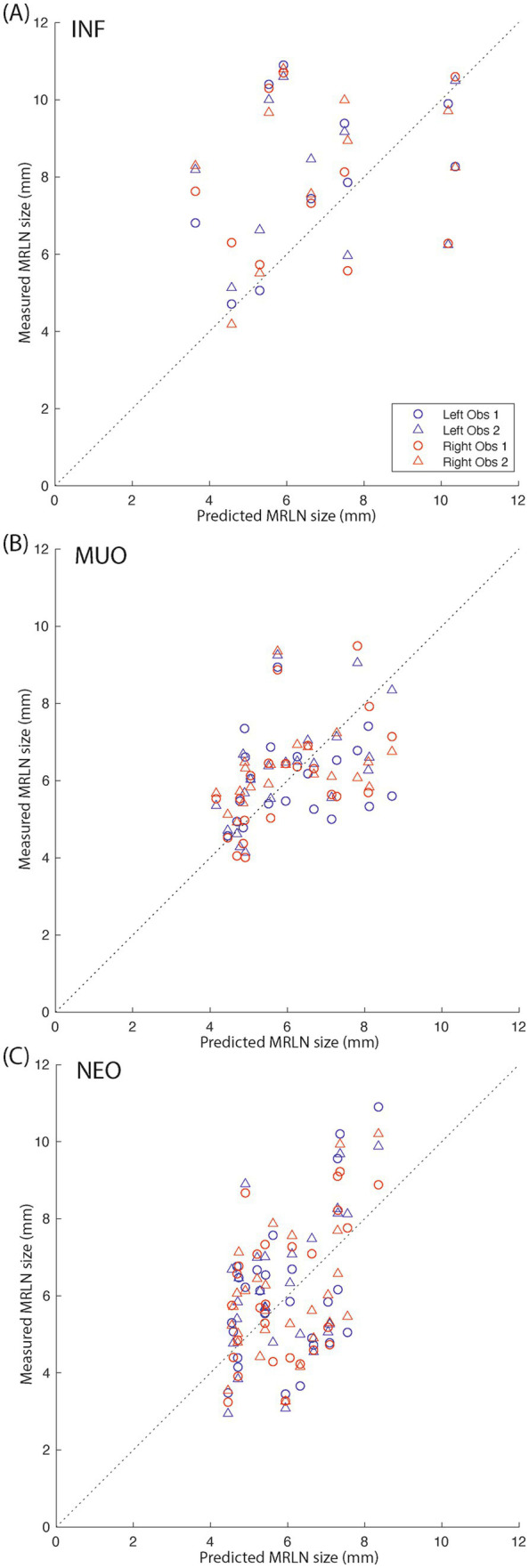
Measured vs. predicted lymph node size for affected subjects. **(A)** Infectious subjects; **(B)** subjects with MUO; and **(C)** subjects with intracranial neoplasia. The dotted black lines indicate y = x. Blue markers indicate left medial retropharyngeal lymph nodes, and red markers indicate right medial retropharyngeal lymph nodes. Circles indicate observer 1 measurements, and triangles indicate observer 2 measurements.

We then predicted the expected size of the left and right MRLNs in affected subjects, using the age and weight of the affected subjects as input to the linear regression model created using control subject data. The median predicted measurement and range are shown alongside the measured values for each affected subgroup in Table 3. Figure 8 shows the relationship between measured and predicted lymph node size, separated by affected subgroup. The median value of the difference between measured and predicted lymph node size was not different from zero for any subgroup.

## 4 Discussion

The measured maximum short-axis diameter of the MRLNs in dogs using T2 spin-echo transverse images acquired on 3T MRI had similar minimum values but higher maximum values than those previously reported for CT (7). In contrast, the range of measured values was slightly narrower than those reported sonographically (9). These measurements were similar between the two independent observers.

For dogs with structurally normal brains, body weight and age had a modest relationship to MRLN size, consistent with previous reports using ultrasound (9). The relationship between age, weight, and lymph node size was generally well-represented by a generalized mixed-effects linear regression model for all but the largest lymph nodes, which were associated with the largest and youngest dogs. This discrepancy may be due to the small number of control subjects within this demographic in our sample or could indicate a more complex interplay between body weight and the maturation of MRLNs.

Compared to a reference range based on dogs with normal head/brain MRI and normal CSF tap, dogs with a diagnosis of infectious brain disease, MUO, or primary CNS neoplasia had similar MRLN size. This is likely due to the wide reference range of lymph node sizes for control subjects. There were age- and weight-related differences among the subjects in the affected subgroups, consistent with previously reported demographic characteristics associated with disease risk. When a linear model designed to account for age- and weight-related variability in lymph node size was used to predict MRLN size in dogs with structural brain disease, small systematic differences were seen between predicted and measured sizes, though these did not reach significance. Specifically, the model tended to underestimate lymph node size for dogs with infectious brain disease. However, these dogs were also significantly younger (and slightly larger) than the control subject sample, and the model is demonstrated to underestimate lymph node size in such cases, regardless of disease state. Therefore, we conclude that MRLN size is not consistently larger in dogs with structural infectious brain disease than in control subjects.

Limitations of this study include its retrospective nature and small sample size. Due to variations in dog head size and shape, medial retropharyngeal lymph nodes are not routinely included in the evaluation of the canine brain, so few available studies met our inclusion criteria. Given the correlation, though modest, between lymph node size and subject body weight and age, more accurate reference ranges for normal canine medial retropharyngeal lymph node size could be generated from data stratified by these variables. However, our control sample is too small to allow for useful estimation of normal population parameters in subgroups; furthermore, the between-subject variability in lymph node size is relatively large compared to the effects attributable to age and body weight, so reference ranges are expected to remain relatively broad even if these factors can be accounted for. An additional concern is that the control sample may not accurately reflect a truly normal population of dogs, given that these dogs underwent brain MRI due to clinical findings consistent with neurological disease.

Within the limits of this study, MRLN size in dogs, measured as M_SATD on transverse T2 spin-echo 3T MRI, is similarly estimated by independent observers, depends mildly on body weight and age, and cannot be used to prioritize differentials for structural brain disease. Despite the failure of this study to reject the null hypothesis, we believe there are several avenues available for future investigation of the relationship between intracranial disease lymphatics of the head and neck. Size is just one parameter that may indicate lymphadenopathy; evaluation of additional imaging parameters (such as the pattern of contrast enhancement post-gadolinium), though potentially more difficult to standardize between observers, may be more sensitive markers of lymph node disease. Given high inter-individual variability, a within-subject comparison design, such as the evaluation of lymph node size in the same individuals in diseased (pre-treatment) vs. healthy (post-treatment) states, may also be better suited to detect small changes in lymph node size. Finally, direct sampling of the medial retropharyngeal lymph nodes, either premortem (such as under ultrasound guidance) or on postmortem histology, may provide a complementary method to detect changes in the cellular makeup of these tissues in association with different intracranial disease states.

## Data Availability

The raw data supporting the conclusions of this article will be made available by the authors, without undue reservation.

## Footnotes

^1^MAGNETOM Verio, Siemens Medical Solutions USA, Inc., Malvern, PA.

^2^MATLAB R2022a Update 2, maci64, MathWorks, Natick, MA.

^3^MedCalc Statistical Software version 22.023, MedCalc Software, Ltd., Ostend, Belgium.

## References

[1] Chae J Choi M Choi J Yoo SJ. The nasal lymphatic route of CSF outflow: implications for neurodegenerative disease diagnosis and monitoring. Anim. Cells Syst. (2024) 28:45–54. doi: 10.1080/19768354.2024.2307559 PMC10826790 38292931

[2] Johnston M Zakharov A Papaiconomou C Salmasi G Armstrong D. Evidence of connections between cerebrospinal fluid and nasal lymphatic vessels in humans, non-human primates and other mammalian species. Cerebrospinal Fluid Res. (2004) 1:2. doi: 10.1186/1743-8454-1-2, PMID: 15679948 PMC546409

[3] Belz GT Heath TJ. Lymph pathways of the medial retropharyngeal lymph node in dogs. J Anat. (1995) 187:517–26. PMID: 7559125 PMC1167010

[4] Leeds SE Kong AK Wise BL. Alternative pathways for drainage of cerebrospinal fluid in the canine brain. Lymphology. (1989) 22:144–6. PMID: 2601407

[5] Johnson PJ Elders R Pey P Dennis R. Clinical and magnetic resonance imaging features of inflammatory versus neoplastic medial retropharyngeal lymph node mass lesions in dogs and cats. Vet Radiol Ultrasound. (2016) 57:24–32. doi: 10.1111/vru.12288, PMID: 26346524 PMC7169271

[6] Kneissl S Probst A. Magnetic resonance imaging features of presumed normal head and neck lymph nodes in dogs. Vet Radiol Ultrasound. (2006) 47:538–41. doi: 10.1111/j.1740-8261.2006.00182.x, PMID: 17153061

[7] Belotta AF Sukut S Lowe C Waldner C Randall EK MacDonald VS. Computed tomography features of presumed normal mandibular and medial retropharyngeal lymph nodes in dogs. Can J Vet Res. (2022) 86:27–34. PMID: 34975219 PMC8697330

[8] CLSI. Defining, establishing, and verifying reference intervals in the clinical laboratory. Pennsylvania: Clinical and laboratory standards institute (2016).

[9] Ruppel MJ Pollard RE Willcox JL. Ultrasonographic characterization of cervical lymph nodes in healthy dogs. Vet Radiol Ultrasound. (2019) 60:560–6. doi: 10.1111/vru.12784, PMID: 31313406

